# Nurses practice towards palliative care in Shire Endasilasie health facilities, Northern Ethiopia: a cross-sectional study

**DOI:** 10.11604/pamj.2020.35.110.18648

**Published:** 2020-04-08

**Authors:** Teklay Zeru, Hadgu Gerensea, Hagos Berihu, Mebrahtom Zeru, Tewolde Wubayehu

**Affiliations:** 1 Department of Pediatric Nursing, School of Nursing, College of Health Science and Comprehensive Specialized Hospital, Aksum University, Tigray, Ethiopia; 2 Department of Maternity and Reproductive Health, School of Nursing, College of Health Science and Comprehensive Specialized Hospital, Aksum University, Tigray, Ethiopia; 3 Department of Biomedical Science, College of Health Sciences, Adigrat University, Adigrat, Tigray, Ethiopia; 4 Department of Pediatrics, School of Medicine, College of Health Science and Comprehensive Specialized Hospital, Aksum University, Tigray, Ethiopia

**Keywords:** Palliative care, practice, governmental health facility, Shire Endasilasie

## Abstract

**Introduction:**

The public health strategy of the World Health Organization for palliative care is to increase access to palliative care services by integrating it with the healthcare systems. Therefore, the value of palliative care service provision by nurses who deliver the majority of care to chronical patients is an important issue. The objective of the study is assessing nurses' practice of palliative care.

**Methods:**

A facility based cross-sectional study was carried out among 278 nurses working in governmental health facilities of Shire Endasilasie town, Tigray region, Ethiopia from February to June 2018. The questionnaire was revised based on the findings of the pre-test. The collected data was checked for its completeness, consistency, and accuracy before analysis. Data were entered and analyzed using SPSS version 22. The final result was reported using text and tables.

**Results:**

A total of 278 nurses were included in the study and the response rate was 100%. The majority of the participants (71.9%) were females and the mean age of the respondents was 32.08 years (range from 20 to 60). Approximately two-thirds (74.8%) of the respondents had poor knowledge of palliative care practice. Half of the study participants reported emotional support gained as primary psychological support. Commonly used drugs for severe pain were paracetamol or ibuprofen 202 (72.2%) and 47.8% nurses focus on quality patient pain assess.

**Conclusion:**

The majority of the nurses had a poor practice of palliative care.

## Introduction

Worldwide, around ten million people are suffering by life-threatening illnesses such as HIV/AIDS and cancer [1]. The majority of the cases occur in developing countries where quite often there is inadequate access to prompt and effective treatment for such problem [2]. The implementation of palliative care (PC) service using effective, low-cost approaches is the best alternative to respond to the urgent needs of the patient with a terminal illness and improve quality of life for those with chronic illnesses [1]. The goals of palliative care can and should be incorporated into everyday practice [3,4]. As currently used in America medicine, “palliative care” is becoming a widely accepted term for an approach to the management of terminally ill patients that focuses on symptom control and support rather than cure or life prolongation [5]. American quality forum is dedicated to improving the quality of care and focuses on palliative care recognizing its growing place within the broader scope of health care [6]. Discussion between the importance of clinical activities or the issue that supportive care is more important will remain a central issue [7,8]. Palliative care is a philosophy of care and therefore it can be delivered in a variety of settings, including institutions such as hospitals, inpatient hospice and home care for older people which constitutes specialty palliative care and hospice care varies both within and across a country [9,10].

Every year nearly half a million people die of cancer in sub Saharan Africa. Despite the rapid improvement of palliative care for cancer, the expansion of the service is still low [11]. The WHO public health strategy for palliative care aims to increase access to palliative care services through its integration into healthcare systems [1]. Studies of hope in palliative care, over the past ten years, have focused primarily on those individuals in the advanced stages of cancer and the human immunodeficiency virus [12]. Studies using quantitative methodology have focused on exploring hope levels across the dying trajectory and the relationship between hope and other psychosocial variables while those using qualitative methodology have focused on the meaning of hope and elucidating how terminally ill individuals maintain and engender their hope [13]. Therefore, the value of palliative care to nurses who deliver a large proportion of the care to chronically ill patients is unquestionable [14] and there is a need to help and educate nurses for the provision of palliative care service and care for terminally ill patients. So, the first step in developing a strategy to help and educate nurses about palliative care is to assess the current practice as there is limited research on palliative care [15]. The aim of this study was to assess nurses' practice towards palliative care among nurses working in governmental health facilities in Shire Endasilasie town, Tigray regional state, Ethiopia.

## Methods

### Study area

The study was carried out in Shire Endasilasie town which is 1084 km away from the capital city of Ethiopia, Addis Ababa and 304 km from the capital city of Tigray regional state, Mekelle. There were four government health facilities in Shire Endasilasie town (two health centers and two hospitals). The study was conducted from February to June 2018. An institution-based cross-sectional study was used to assess nurses' palliative care practice. All nurses working in governmental health facilities in Shire Endasilasie town and all sampled nurses working in medical, surgical, pediatrics, and neonatal wards, intensive care unit (ICU), outpatient and emergency departments were included. Nurses engaged in the care of patients with chronic illness and/or terminal illness, nurses deployed to sterilization department, central triage and on leave were excluded from the study.

The sample size was calculated using a single population proportion formula as follows:

$$n=\frac{\left(Z\text{α}/2)\;2\text{p}\;(1-\text{p}\right)}{d^{2}}$$

Where n = minimum sample size required for the study, d = margin of error = 0.05, Zα / 2 value of standard normal distribution (z = 1.96) with confidence interval of 95% and α was 0.05. Previously in a study, 76.2% had poor knowledge of palliative care practice [16],

n = (1.96)^2^ 0.762 (1 - 0.762) / (0.05)^2^ = 278

The study participants were selected proportionally from nursing staffs each of the four governmental health facilities (Omar health center, Alganesh health center, Central defense hospital and Suhul hospital) were selected proportional to a number of nurses in the selected facilities. The study participants from each health facility were selected using a systematic sampling technique using the nurses' registry in the respective health facility. The first sample was selected by lottery method.

### Data collection

Data were collected by self-administering the practice questions adopted from the literature of similar studies [16-20]. The questionnaire was prepared in English and then translated into the local language which is Tigrigna and then back to English by different people and corrected accordingly. A pre-test was done on 10% (28) of the nurses in Kahsay Abera hospital which is nearest to Endasilasie Shire. For the purpose of validity and reliability issues, the questionnaire was revised based on the findings of the pre-test. The collected data was checked for its completeness, consistency, and accuracy before analysis. Data were entered and analyzed using SPSS version 22. Descriptive statistics were presented using tables.

### List of operational definition

**Palliative care:** palliative care is an approach that improves the quality of life of patients and their families facing the problems associated with a life-threatening illness, through the prevention and relief of suffering by means of early identification and impeccable assessment and treatment of pain and other problems, physical, psychosocial and spiritual [1].

**Good palliative care practice:** ≥75% of total knowledge aspect of practice questions.

**Poor palliative care practice:** <75% of the total practice questions.

## Results

A total of 278 study participants were included in the study and the response rate was 100%. The number of study participants from Umer health center, Alganesh health center, Central defense hospital, and Suhul hospital was 35(12.58%), 40(14.29%), 82(29.50%) and 121(43.52%) respectively. The majority of the participants (71.9%) were females and the mean age of the respondents was 32.08 years ± 6.25 SD (range from 20 to 60 years of age). The respondents from medical wards, surgical wards and pediatrics wards were 55(19.8%), 68(24.46%), 45(16.18%) respectively. The neonatology unit, ICU and emergency department constitute 18(6.4%), 20(7.1%), out patient department (OPD) 50(18%) of the study participants respectively (Table 1).

**Table 1:** Socio-demographic characteristics of nurses at shire town health facilities in Tigray region, Ethiopia, June 2018

Characteristics		Frequency	Percentage
		No (278)	% (100)
**Work institution**	Omar health center	35	12.58
	Alganesh health center	40	14.39
	Central defense hospital	82	29.50
	Suhul hospital	121	43.52
**Age of nurses**	20-30	129	46.4
	31-40	76	27.33
	41-50	61	21.94
	50+	12	4.3
**Educational level**	Diploma	120	43.1
	Degree	158	57.9
**Ward/work area**	Medical ward	55	19.8
	Surgical ward	68	24.46
	Pediatrics	45	16.18
	Neonatology	18	6.4
	ICU	20	7.1
	OPD	50	18
	Emergency	14	5.03
	Other	8	2.87
**Work experience**	Less than 5 years	120	43.1
	5-10 years	75	27
	10-15 years	64	23
	Greater than 15 years	19	6.8
**Experience in caring terminally ill patient**	Daily	115	41.3
	Once per week	62	22.3
	Never	54	19.4
	Few times per year	25	8.99
	Once per month	22	8

### Knowledge aspect of the practice of nurses towards palliative care

Approximately two thirds (74.8%) of the respondents had poor knowledge of palliative care. Only 27.2% of the nurses initiated palliative care discussions with patients during diagnosis while 27.0% of the nurses did as the disease progressed and 20.9% of the respondents did inform terminally ill patients about their diagnosis. A small proportion (27.1%) of the nurses did inform terminally ill patients about their diagnosis and concerning addressing psychological support, 139(50.0%) of the respondents reported emotional support gained and 57(20.5%) hiding the reality. Regarding patient pain assess, 133(47.8%) of them focuses on quality (Table 2).

**Table 2:** Practice of nurses towards palliative care at Shire Endasilasie governmental health facilities, June 2018 (n=278)

No	Characteristics	Multiple responses	Frequency	Percentage
			Yes n (%)	No n (%)
1	Time of palliative care discussion with clients:	During diagnosis	25(27.2%)	67(72.8%)
		When the disease progress	27(27.0%)	73(73.0%)
		At the end of life	18(20.9%)	68(79.1%)
2	Do you inform a terminally ill patient about their diagnosis	Yes	16(27.1%)	43(72.9%)
		No	25(32.9%)	51(67.1%)
		Depending on a family's wish	14(18.9%)	60(81.1%)
		Inapplicable	15(21.7%)	54(78.3%)
3	Factors considered when dealing with a terminally ill patient:	Spiritual	23(27.1%)	62(72.9%)
		Medical situation	27(27.0%)	73(73.0%)
		Cultural	12(19.7%)	49(80.3%)
		Psychological	8(25.0%)	24(75.0%)
4	How do you address spiritual issue:	Connect with spiritual counselor	19(23.8%)	61(76.3%)
		Listen with empathy	15(26.8%)	41(73.2%)
		Impose your own view	24(26.1%)	68(73.9%)
		Understand patient reaction	12(24.0%)	38(76.0%)
5	Cultural assessment during patient care should include:	Truth-telling and decision making	11(20.8%)	42(79.2%)
		Preference regarding disclosure of information	21(28.0%)	54(72.0%)
		Dietary preference	19(31.7%)	41(68.3%)
		Language, family communication	15(23.8%)	48(76.2%)
		Perspective on death, suffering & grieving	4(15.4%)	22(84.6%)
6	How you address psychological:	Emotional support	36(25.9%)	103(74.1%)
		Counseling the patient	21(25.6%)	61(74.4%)
		Hiding the truth	13(22.8%)	44(77.2%)
7	Who is responsible for decision making?	Patient	24(21.2%)	89(78.8%)
		Family	16(23.9%)	51(76.1%)
		My own	26(31.3%)	57(68.7%)
		Another health professional	4(26.7%)	11(73.3%)
8	What is your concern about terminally ill patient care?	Patient right	17(24.6%)	52(75.4%)
		Treat	18(26.9%)	49(73.1%)
		Doubting your professionalism	26(25.7%)	75(74.3%)
		Attention seeking behavior	9(22.0%)	32(78.0%)
9	Communication with the family of the terminally ill patient depends on:	Family’s ability to assimilate	25(26.0%)	71(74.0%)
		Their involvement in decision making	30(23.3%)	99(76.7%)
		Your willingness to disclose information	15(28.3%)	38(71.7%)
10	Which medication do you use in your practice for severe pain?	Paracetamol/Ibuprofen	55(27.2%)	147(86.5%)
		Codeine	10(25.6%)	29(74.4%)
		Morphine	5(13.5%)	32(72.8%)
11	How do you assess patient pain?	Grade with face	15(29.4%)	36(70.6%)
		Intensity	21(36.2%)	37(63.8%)
		Location	3(8.3%)	33(91.7%)

## Discussion

The finding of this study showed that the majority (74.8%) had a poor practice which is similar to the result from New Heaven [21]. Poor palliative care practice in this result could be related with respondents' poor knowledge on the aspect of PC practice and it might also be due to the fact that study subjects had doubts on professionalism which affects the PC practice habit. Nearly one fourth of participated nurses did not inform terminally ill patients about their diagnosis which was lower compared to studies done in Lebanon, United States, England and Addis Ababa [16,22-24] and lower from study in Norway [16,18], since nowadays diagnosis of patients used to be expressed by nurses may not challenge to disclose to patients in the case of Ethiopia. The finding of this study reveals that more than one-third of the respondents consider medical treatments and 30.6% prefer spiritual other than cultural and psychological beliefs when treating the end of life patients. This finding is similar to the study done in Lebanon [20]. This could be due to Ethiopians introducing modern medicine and the availability of health facilities near the population as well as providing great value and concern for religion. Twenty-seven percent of cultural assessment during patient care preference regarding disclosure of information. On the contrary, the study done in America reported that majority of nurses viewed that truth-telling and decision making is practiced [25]. Half of the nurses in this study addressed the psychological issues of the patient with emotional support. On the contrary, the study done in Norway reported that the majority of nurses viewed that lying to the patients about their diagnosis and prognosis as unethical [26]. Is also the difference from this Addis Ababa majority nurses were hiding the truth [16]. Nearly three fourth (72.7%) of the respondents' use paracetamol or ibuprofen for chronic pain management. This might be a result of severe side effect of opioid analgesics and/or nurses are not recommended to prescribe opioid analgesics. Similarly, in a study done in Malawi, health workers required access to pain medication and knowledge of oral morphine in order to provide appropriate patients care [27]. This is important because the treated pain has a bad feeling on the patient, limitation of activity in daily living and social interaction [28]. With estimation, half of nurses concern on the quality of pain assessment. In contrast, grade with the face is the priority of nurses [17].

## Conclusion

The majority of the nurses had poor knowledge of the practical aspects of palliative care. Guidelines for nurses' palliative care practice should be developed and distributed to all health facilities.

### What is known about this topic

- The majority of nurses had a poor practice of palliative care;
- Palliative care was provided only to patients with cancer and HIV;
- The perception of terminally ill patients concern as need of treatment.

### What this study adds

- Approximately two thirds (74.8%) of the respondents had poor practice towards palliative care;
- Only a few nurses initiated palliative care discussions with patients during diagnosis;
- Palliative care includes the management of patient pain and family members.

## Competing interests

The authors declare no competing interests.
